# Brain and muscle chemistry in myalgic encephalitis/chronic fatigue syndrome (ME/CFS) and long COVID: a 7T magnetic resonance spectroscopy study

**DOI:** 10.1038/s41380-025-03108-8

**Published:** 2025-07-12

**Authors:** Beata R. Godlewska, Amy L. Sylvester, Uzay E. Emir, Ann L. Sharpley, William T. Clarke, Stephen R. Williams, Ana Jorge Gonçalves, Betty Raman, Ladislav Valkovič, Philip J. Cowen

**Affiliations:** 1 Clinical Psychopharmacology Research Unit, Department of Psychiatry, University of Oxford, Oxford, UK; 2 Oxford Health NHS Foundation Trust, Oxford, UK; 3 Wellcome Centre for Integrative Neuroimaging, University of Oxford, Oxford, UK; 4 University of North Carolina, Department of Radiology, Chapel Hill, USA; 5 Division of Informatics, Imaging and Data Science; Faculty of Medicine, Biology and Health; University of Manchester, Manchester, UK; 6 Wolfson Molecular Imaging Centre, Faculty of Biology, Medicine and Health, University of Manchester, Manchester, UK; 7 Division of Cardiovascular Medicine, Radcliffe Department of Medicine, University of Oxford, Oxford, OX3 9GP UK; 8 Oxford Centre for Clinical Magnetic Resonance Research (OCMR), Division of Cardiovascular Medicine, Radcliffe Department of Medicine, University of Oxford, Oxford, UK; 9 Department of Imaging Methods, Institute of Measurement Science, Slovak Academy of Sciences, SVK, Bartislava, Slovakia

**Keywords:** Neuroscience, Diseases

## Abstract

Myalgic encephalitis/chronic fatigue syndrome (ME/CFS) is a common debilitating medical condition, whose main symptoms - fatigue, post-exertional malaise and cognitive dysfunction – are also present in many cases of long COVID. Magnetic resonance spectroscopy (MRS) allows the insight into their pathophysiology through exploration of a range of biochemicals putatively relevant to aetiological processes, in particular mitochondrial dysfunction and energy metabolism. 24 patients with ME/CFS, 25 patients with long COVID and 24 healthy controls (HC) underwent brain (pregenual and dorsal anterior cingulate cortex, respectively, pgACC and dACC) and calf muscle MRS scanning at 7 Tesla, followed by a computerised cognitive assessment. Compared to HC, ME/CFS patients had elevated levels of lactate in both pgACC and dACC, while long COVID patients had lowered levels of total choline in dACC. By contrast, skeletal muscle metabolites at rest did not significantly differ between the groups. The changes in lactate in ME/CFS are consistent with the presence of energetic stress and mitochondrial dysfunction. A reduction in total choline in long COVID is of interest in the context of the recently reported association between blood clots and ‘brain fog’, and earlier animal studies showing that choline might prevent intravascular coagulation. Importantly, differences in findings between ME/CFS and long COVID suggest that the underlying neurobiological mechanisms, while leading to similar clinical presentations, may differ. An important implication is that patients with ME/CFS and those with fatigue in the course of long COVID should not be studied as a single group, at least until the mechanisms are better understood.

## Introduction

Myalgic Encephalomyelitis/Chronic Fatigue Syndrome (ME/CFS) is a persistent and debilitating medical condition, affecting around 1% of the population [1]. Its core symptoms are fatigue not caused by exertion and not alleviated by rest, post-exertional malaise (PEM) and cognitive dysfunction [2]. Similar symptoms are present in long COVID, a long-term sequela of the acute infection with SARS-CoV-2 virus (COVID-19), where fatigue is one of the most common symptoms [3, 4]. Interestingly, both ME/CFS and long COVID are triggered by a virus. There are however some important differences between the conditions in terms of their putative causative factors. Long COVID is triggered exclusively by SARS-CoV-2 virus, while ME/CFS has been related to a number of viruses, most commonly EBV but also other viral infections, such as Ross River virus or other herpes viruses [5]. Also, in some cases, ME/CFS is triggered by non-viral factors, such as emotional stress, major life events and physical trauma, which is not the case for long COVID [5]. Some other important differences between ME/CFS and long COVID need to be acknowledged. An array of additional physical symptoms is commonly present in long COVID [3]. There is also a striking difference in the time for which both diseases have been present in the population and how long individuals have suffered from them. Thus, SARS-CoV-2 is a new virus for humans while EBV is an ancient virus that co-evolved with humans. These differences may result in subtle differences in these and other chronic post-viral syndromes. Despite relatively high prevalence of both conditions and their debilitating impact on life (for example, one-quarter of ME/CFS patients are bed- or house-bound) [6], the understanding of mechanisms leading to the development of fatigue is poor. This has important clinical consequences, such as the lack of biological markers [7] and the hindered development of targeted pharmacological treatments.

While acknowledging the differences between ME/CFS and long COVID, the similarities between fatigue related symptoms are intriguing and raise a question about potential shared mechanisms [8]. Some of the common symptoms of both ME/CFS and long COVID, such as cognitive impairment, disturbed sleep and altered pain sensitivity, are most likely of central origin. However, in many cases peripheral symptoms, such as muscle fatigue, either prevail or are an important part of the clinical presentation. It is yet unclear whether central and peripheral symptoms are linked or develop independently of each other, hence a better understanding of their connections is important. One way of achieving this is with the use of magnetic resonance spectroscopy (MRS). MRS is a tool, which through exploiting behaviours of metabolites in the magnetic field, allows an in vivo assessment of chemistry of the body organs, including the brain and muscle [9].

Early brain studies using proton (^1^H) MRS at low 1.5 Tesla (1.5T) field strengths in ME/CFS reported increased levels of choline containing compounds (tCho) [10, 11], a marker of cell membrane turnover [12]. This finding was corroborated by a 3T Magnetic Resonance Spectroscopic Imaging (MRSI) study, assessing multiple regions in the brain [13]. MRS work at 3 Tesla (3T) from one research group demonstrated elevated levels of ventricular lactate in cerebrospinal fluid (CSF) [14–17], as well as a reduction in the major endogenous free radical scavenger, glutathione (GSH) [16, 18]. Increased lactate was also found in multiple brain regions in the aforementioned MRSI study [13]. As far as we know, the only research at ultra-high field strength of 7T is the investigation carried out by our group where we found decreased levels of GSH, and creatine in ME/CFS [19]. Taken together, the above findings are supportive of the hypotheses of increased oxidative stress, inflammation and impaired oxidative energy metabolism in ME/CFS.

MRS brain studies in long COVID are still scarce. Importantly, fatigue is not often a point of focus. Indeed, there was only one investigation in which the presence of fatigue (for at least 3 months) constituted the main and necessary inclusion criterion. This study, performed at 1.5T, showed a decrease in creatine, consistent with abnormal energy metabolism [20]. Another study, conducted at 3T, showed an association between elevated Glx concentrations and widely defined ‘brain fog’ [21]. The results of long COVID investigations cautiously suggest that impaired metabolism and neuronal dysfunction may play a role in mental fatigue, similar to hypotheses for ME/CFS. While there are no reports of changes in MRS markers of oxidative stress, its role in long COVID gets support from studies showing elevated oxidative stress blood biomarkers [22]. As far as we know, there are no MRS studies at 7T in long COVID.

MRS can also be used to explore the mechanisms underlying peripheral fatigue. ^1^H MRS in the muscle allows detection of multiple metabolites, of which creatine, acetyl-carnitine, carnosine and intramyocellular lipids (IMCL) have been shown to be useful biomarkers of muscle metabolism [23]. ^1^H MRS studies in ME/CFS and long COVID are however still rare, in fact we are aware of only one, showing decreased creatine concentration in long COVID, which correlated inversely with muscle pain [20].

The main aim of this study was to replicate our initial findings of decreased creatine levels in ME/CFS consistent with abnormal energy metabolism [19]. Additionally, we explored whether such abnormalities could also be seen in the muscle, and whether there is a relationship between changes in the brain (central origin of symptoms) and in muscle (peripheral origin of symptoms). An inclusion of long COVID participants with predominant fatigue allowed an exploration of such changes in a different condition where fatigue is one of the main symptoms. Due to our interest in energy processing in the brain, we aimed to assess levels of lactate, which is either a waste product of alternative energy processing pathways or an additional source of energy delivered to the brain when the usual ways of energy production fail [24]. Because of the hypothesised abnormalities in oxidative stress and suggested links between initial inflammation, glutamate excitotoxicity and neuronal dysfunction/loss [25], our additional interest was in, GSH, glutamate and glutamine, and total choline as biomarkers of these processes. We used MRS at 7T ultra-high field strength, which offers a unique opportunity for more precise quantification of a range of neurometabolites due to greater signal to noise ratio (SNR) and increased spectroscopic resolution compared to 3T [9, 26]. Given the importance of cognitive dysfunction (experience as ‘brain fog’) in both ME/CFS and long COVID, we also explored the relationship between cognitive function and changes in brain biochemistry that may underlie cognitive deficits.

## Methods

### Ethics approval and consent to participate

The study obtained ethical approval from the National Research Ethics Service Committee (NRES), South-Central Oxford B (reference number 19/SC/0603). Written informed consent was obtained from all participants. Written informed consent for publication of non-identifiable brain images from the study was obtained from all participants. All methods were performed in accordance with relevant guidelines and regulations.

### Participants, clinical ratings and cognitive function

In total, there were 73 participants, including 24 patients with ME/CFS (16 females, 8 males, mean age 32.6 years, range 19–49 years); 25 patients with long COVID (19 females, 6 males, mean age 44.3, range 27–67); and 24 healthy volunteers (12 females, 12 males, mean age 40.5 years, range 30–65 years). ME/CFS and HC sample size replicated our pilot study [19]. All participants gave full informed written consent and underwent an MRS scan and a computerised cognitive assessment. Two additional ME/CFS patients had to be withdrawn from the scan due to adverse effects - a panic attack shortly after entering the scanner and a heating sensation - no MRS and cognitive data were obtained for these participants.

Participants were recruited from the local community through local newspapers and social media advertisements, support groups, flyers in long COVID and ME/CFS care clinics between February 2020 and July 2022. Recruitment for ME/CFS patients started just before COVID related restrictions were introduced and research was suspended: one patient and one healthy control were recruited in February 2020, others from February 2021. ME/CFS diagnosis was made by an appropriate professional and also met Center for Disease Control and Prevention (CDC) criteria for ME/CFS [27], which were administered by a clinically trained member of the team (BRG). Long COVID participants had the diagnosis initially made by a clinician based on the clinical history and the presence of typical acute COVID-19 symptoms (at least two of the following: high fever, shortness of breath, persistent cough, loss of taste or smell). Due to recruiting patients who had an acute infection as early as in February 2020, 12 participants did not have laboratory confirmation of SARS-CoV-2 infection as appropriate tests were not available at the time of infection. All long COVID participants were required to have new fatigue following COVID-19 infection (i.e. absent before infection), persisting for at least 12 weeks since infection, present at the time of assessment, and impairing everyday functioning. Exclusion criteria for all groups included current or previous psychiatric comorbidities as diagnosed by DSM-5, using a structured interview (The Structured Clinical Interview for DSM-5, SCID-5) [28] if deemed relevant by the investigator, any general medical conditions known to influence energy processing and/or linked to fatigue, current treatment with any medication/supplementation likely to interfere with energy metabolism if deemed relevant by the investigator, contra-indications to magnetic resonance (MR) imaging, pregnancy or breast feeding. Long COVID and healthy participants were excluded if they had previous history of ME/CFS.

Fatigue was measured with the Chalder Fatigue Scale (ChFS) [29]. As depression has been previously shown to be associated with changes in neurometabolites, mood and anxiety ratings were performed: the Beck Depression Inventory II (BDI-II) [30] for mood, the Spielberger State Anxiety Inventory (STAI) for anxiety [31].

Cognitive function was evaluated with the Brief Assessment of Cognition in Schizophrenia (BACS) battery of cognitive tests, which allows a measurement of global cognitive function across six domains: verbal memory with word list learning task, working memory with digit sequencing task, verbal fluency with controlled oral word association task, motor function with token motor task, attention and speed of information processing with symbol coding task and executive functions with Tower of London task. The scores on BACS subtests comprise a composite score. Scores are corrected for age and gender using stratified general population norms and expressed as a z-score [32].

### Magnetic resonance spectroscopy

**Fig. 1:**
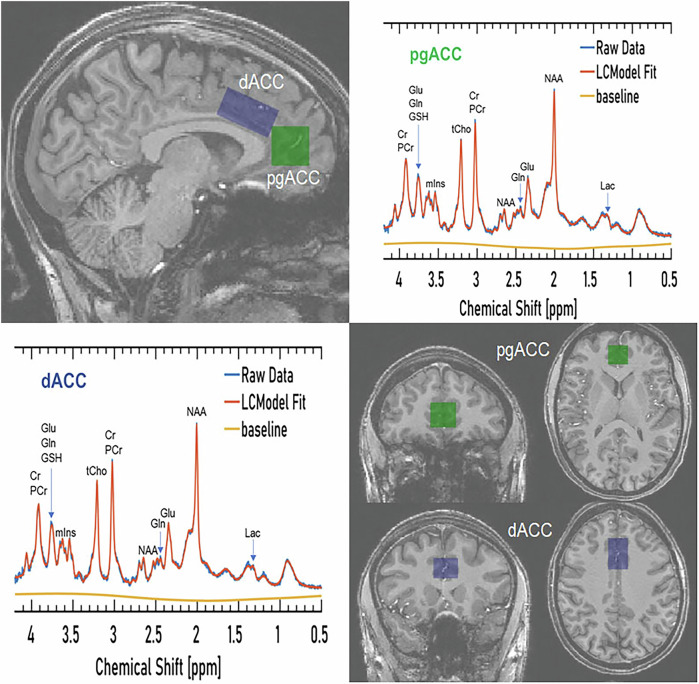
Voxel placement and representative spectra from the pregenual anterior cingulate cortex (pgACC, green) and dorsal anterior cingulate cortex (pgACC, blue). Lac lactate, tCho total choline, Glu glutamate, Gln glutamine, GSH glutathione, Cr creatine, PCr phosphocreatine, myoIns myo-inositol, NAA N-acetylaspartate.

Participants underwent ^1^H MRS scanning at the Wellcome Centre for Integrative Neuroimaging in Oxford. Scanning was performed on a 7T Siemens MAGNETOM scanner (Siemens, Erlangen, Germany) with a Nova Medical 32 channel receive array head coil. Spectra were measured from a voxel in the pregenual anterior cingulate cortex (pgACC, 20 × 20 × 20 mm) and dorsal anterior cingulate cortex (dACC 20 × 15 × 30 mm) (Fig. 1). These regions were chosen because of their putative role in the neural mechanisms of ME/CFS and long COVID, in particular with regard to the integration of information from various levels [33, 34], with pgACC key for emotional regulation and autonomic integration, and dACC - for cognition [35, 36]. Additionally, changes in the ACC were shown in previous studies [13]. The voxel was positioned manually by reference to 1-mm isotropic T_1_-MPRAGE image (TR = 2200 ms TE = 2.82 ms, TI = 1050 ms). First- and second-order shims were first adjusted by gradient-echo shimming [37]. The second step involved only fine adjustment of first order shims using FASTMAP [38]. Spectra were acquired using a stimulated echo acquisition mode (STEAM, [39]) pulse sequence (TE/TM = 11/32 ms, TR = 5 s, number of transients = 64) with variable power radiofrequency pulses with optimised relaxation delays (VAPOR, [40]) water suppression and outer volume saturation [41]. Unsuppressed water spectra acquired from the same voxel were used to remove residual eddy current effects, to reconstruct the phased array spectra and for reference for quantification. The choice of acquisition method has minimized any potential T_2_ relaxation differences between populations [42, 43].

**Fig. 2:**
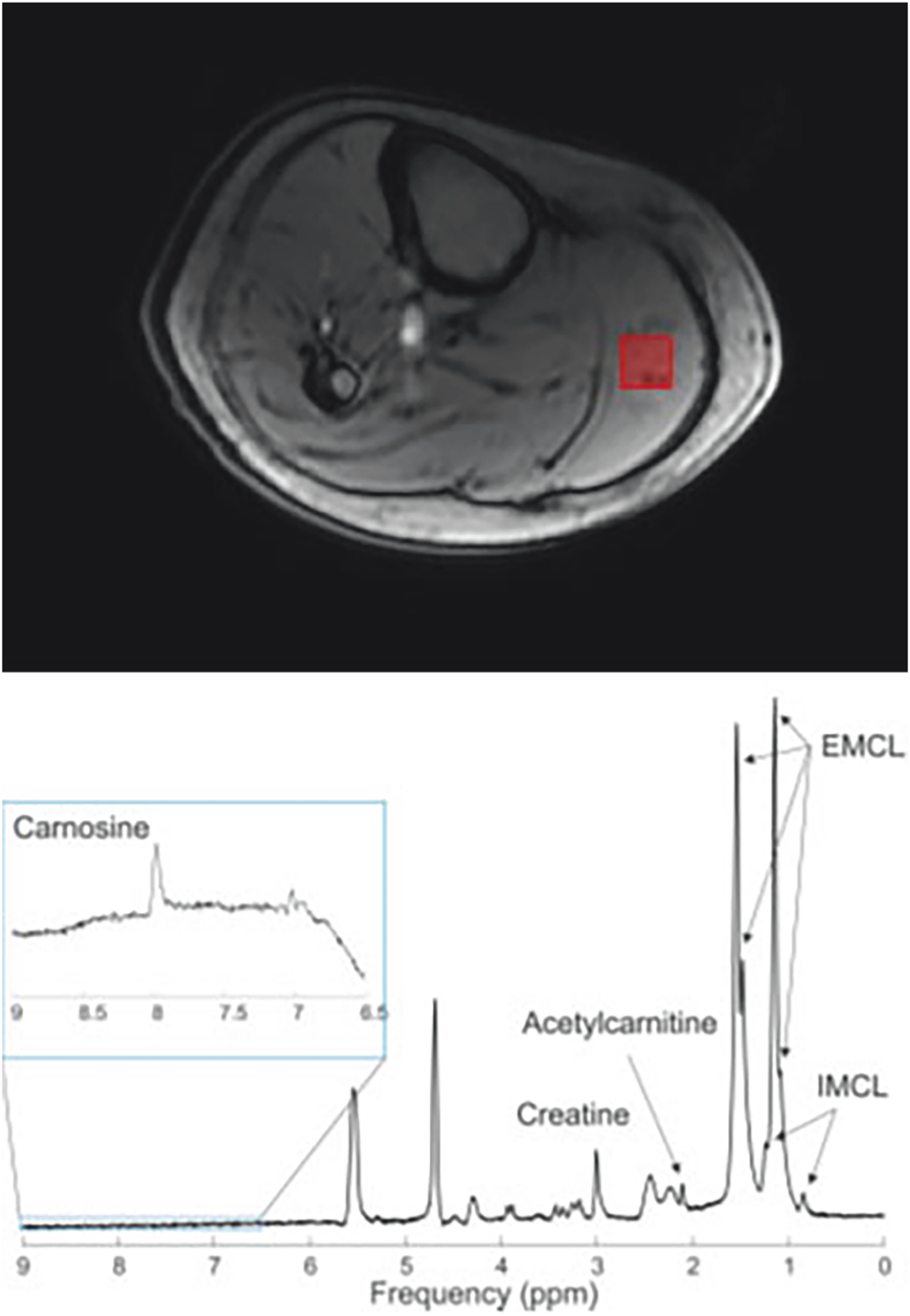
Voxel placement and representative spectra from the calf muscle. IMCL intramyocellular lipids, EMCL extramyocellular lipids.

The muscle data were acquired using a 28 channel knee coil (Quality Electrodynamics [QED], Mayfield Village, USA) or a circular transmit/receive surface coil (10 cm in diameter, Rapid Biomedical, Rimpar, Germany) positioned under the right calf muscle, while the QED coil was unavailable. A STEAM voxel (10 × 10 × 20 mm^3^) was placed inside gastrocnemius medialis muscle avoiding the subcutaneous adipose tissue (Fig. 2). The data acquisition was repeated thrice with different parameters: for carnosine detection (water suppressed spectra with TE = 20 ms, TR = 2.5 s, number of transients = 128) [44]; for intramyocellular and extramyocellular lipids (IMCL and EMCL), creatine (Cr), components containing trimethylamine (TMA) group, and acetyl-carnitine detection (water non-suppressed spectra with TE = 280 ms, TR = 2 s and number of transients = 64) [45–47]; and for water reference acquisition (TE = 20 ms, TR = 2 s and a single transient).

Neurometabolites were quantified with LCModel [48], with prior reported chemical shifts and coupling constants [49, 50] as a basis for the model spectra of aspartate (Asp), ascorbate/vitamin C (Asc), glycerophosphocholine (GPC), phosphocholine (PC), creatine (Cr), phosphocreatine (PCr), γ-amino-butyric acid (GABA), glucose (Glc), glutamine (Gln), glutamate (Glu), glutathione (GSH), myo-inositol (myo-Ins), N-acetylaspartate (NAA), N-acetylaspartylglutamate (NAAG), phosphoethanolamine (PE), scyllo-inositol (scyllo-Ins) and taurine (Tau) were generated based on previously reported chemical shifts and coupling constants.by using GAMMA/PyGAMMA simulation library of VESPA for applying the density matrix formalism (Versatile Simulation, Pulses and Analysis 9). The same RF pulses and sequence timing, like those on the 7-T system, were used to perform simulations. Further, the model spectra included a macromolecule spectrum acquired from the occipital cortex of 5 volunteers, using an inversion recovery sequence (TR  =  3 s, TE  =  11 ms, inversion time TI  =  0.685 s). The concentration of neurometabolites was estimated relative to an unsuppressed water signal obtained from the same VOI [51]. The spectra quality requirement was full width half height linewidth less than 2.5 x standard deviation (SD) above the group mean. All spectra were visually inspected prior to their inclusion in the analysis by a researcher blind to the group allocation. CRLB was not used as the basis for exclusion of individual metabolites [52]. The following parameters were used: echo time (11 ms), T2 of tissue water (40 ms for AttH2O), T2 of metabolite (100 ms for AttMet).

The MP RAGE images were segmented using FSL-FAST [53] and then FSL-MRS [54] to determine grey matter (GM), white matter (WM) and cerebrospinal fluid (CSF) fraction (fGM, fWM, fCSF) in the voxels. The results of segmentations were visually inspected. Concentrations were then corrected for these with the following formula:

$${{\rm{MetCorr}}}=({{\rm{MetConcAbs}}}\times ({{\rm{fGM}}}\times 43300+{{\rm{fWM}}}\times 35880+{{\rm{fCSF}}}\times 55556))/(1-{{\rm{fCFS}}})$$

where [Mcorr] is the corrected concentration, [M] is the water-referenced metabolite concentration from the LCmodel output, fCSF is CSF fraction in the voxel, fGM is GM fraction in the voxel, and fWM is GM fraction in the voxel.

CSF contains very low metabolite concentrations but contributes to the water signal from the region of interest, so it is necessary to correct the metabolite for this apparent dilution effect.

The muscle spectra were acquired from the calf muscle, a region of the body where patients with ME/CFS and long COVID often experience pain. The spectra were analysed using the OXSA toolbox [55], which represents an AMARES [56] implementation in MATLAB (Mathworks, Natick, US). Individual peaks of carnosine, acetyl-carnitine, creatine, TMA, lipids, and water were modelled as single Lorentzians. Lipids surrounding the acetyl-carnitine peak were fitted with a constrained frequency of 2.0–2.1 ppm and 2.17–2.30 ppm to avoid their influence on fitted acetyl-carnitine [46]. Prior to carnosine fitting, the residual water signal and the other metabolite signals were removed using the Hankel-Lanczos Singular Value Decomposition. The spectra quality requirements were full width half height linewidth less than 2.5xSD above the group mean.

The concentration of all metabolites was calculated relative to the unsuppressed water spectrum and expressed as %water signal (IMCL and EMCL) or in absolute concentration values (all other metabolites) calculated using the following formula:

$$[{{\rm{M}}}]=({{{\rm{S}}}}_{{{\rm{M}}}}/{{{\rm{S}}}}_{{{\rm{W}}}})\times ({{{\rm{CF}}}}_{{{\rm{W}}}}/{{{\rm{CF}}}}_{{{\rm{M}}}})\times {{{\rm{n}}}}_{{{\rm{W}}}}/{{{\rm{n}}}}_{{{\rm{M}}}}\times [{{\rm{W}}}]\times {{\rm{W}}} \%$$

Where [M] is the metabolite concentration and [W] is the water concentration (55 M); CF_W_ and CF_M_ are the relaxation correction factors for water and metabolite, respectively; they were determined using block equation based on literature T1 values: (1-e^(-TR/T1))*e^(-TE/T2) [44–46]; n_W_ and n_M_ are the number of protons in a water or metabolite molecule contributing to the signal, e.g., n_W_ = 2; and W% is the approximate water content of skeletal muscle tissue, i.e. 0.7 L/kg wet weight of tissue.

The investigator was blinded to the group allocation during data analysis.

### Statistics

Statistical analyses were performed in SPSS version 25. Differences in metabolite concentrations between patients with ME/CFS, long COVID and healthy controls in pgACC, dACC and muscle were examined using one-way ANOVA, or univariate general linear model where covariates were used; where comparisons between three groups were significant, the same methods were used for comparisons between two groups. As this study was exploratory, there was no correction for multiple testing. Differences were tested using one-way ANOVA for continuous variables and chi-square test for categorical variables. Correlations between individual ratings of fatigue, clinical scores, length of time of illness, and levels of neurochemicals in pgACC and dACC were carried out using Pearson’s product moment and were not corrected for multiple comparisons.

## Results

### Demographics and clinical data

There were no between-group differences in terms of gender (χ2, *p* = 0.158) (see Table 1 for details). One-way ANOVA showed significant differences between the groups in terms of age (*p* = 0.001), hence age was included as a covariate in between group comparisons of metabolite concentrations. The patient and healthy control groups did not differ significantly in terms of BMI (Table 1).

**Table 1:** Demographic data and clinical scores.

	ME/CFS patients [24]	Long COVID patients [25]	Healthy controls [24]	Statistical tests	Independent samples t-tests ME/CFS vs HC Long COVID vs HC ME/CFS vs long COVID
Demographics					
Current age (years)	32.6 (1.9)	44.3 (2.3)	40.5 (2.1)	F = 8.062, *p* = 0.001	0.007
					0.223
					0.001
Gender F/M	16/8	19/6	12/12	χ^2^ = 3.691, *p* = 0.158	N/A
					N/A
					N/A
Age at onset (years)	22.4 (1.6)	42.9 (2.3)	N/A	N/A	N/A
Time since onset (months)	122.5 (20.7)	15.3 (1.6)	N/A	N/A	N/A
Antidepressant medication use	5/24	6/25	N/A	N/A	N/A
BMI	23.4 (0.7)	25.1 (0.9)	22.6 (0.3)	F = 2.973, *p* = 0.058	N/A
					N/A
					N/A
Clinical scales					
ChFS	23.0 (1.1)	23.8 (0.9)	9.4 (0.5)	F = 88.619, *p* < 0.0001	*p* < 0.0001
					*p* < 0.0001
					1.000
BDI	14.2 (1.8)	14.8 (1.4)	2.0 (0.6)	F = 29.184, *p* = 0.000	0.000
					0.000
					1.000
STAI-S	35.3 (1.7)	36.5 (2.4)	29.0 (1.7)	F = 4.276, *p* = 0.018	0.076
					0.026
					1.000

Values represent numbers or mean (SEM).

*F* females, *M* males, *BMI* body mass index, *ChFS* chalder fatigue scale, *BDI* beck depression inventory, *STAI-S* spielberger state anxiety inventory – state. Independent samples t-tests were performed only for significant one-way ANOVA.

As expected, both patient groups had substantially higher ratings of fatigue than controls on Chalder Fatigue Scale (ChFS) (Table 1). Long COVID patients had modestly increased scores on measure of anxiety (STAI), very similar to those of CFS patients; however the latter were not statistically significantly different from controls (Table 1). Both patient groups had higher scores on BDI-II than healthy controls, however. none of the patients met the DSM-5 criteria for major depression, assessed with SCID. Increased BDI-II scores resulted from items related to mental and physical fatigue (tiredness/fatigue, loss of energy, items referring to cognitive symptoms such as concentration difficulties) apart from one patient, who had a higher score across most items, while still not meeting the criteria for depression. All participants were non-smokers. In 20/24 ME/CFS patients, development of fatigue and other symptoms was preceded by a viral infection. Five out of 24 ME/CFS patients and 6 out of 25 long COVID patients were taking antidepressant medications. The list of prescription medications used by individual participants and comorbidities can be found in Supplementary Table 1.

### Cognitive scores

Due to technical problems, we were unable to acquire cognitive data for all the participants. BACS data were available for 18 ME/CFS patients, 25 long COVID patients and 22 healthy controls. The only significant between-group difference in cognitive function observed in this study regarded executive function, i.e. a set of mental skills that help people manage everyday tasks and solve problems requiring planning, flexibility in thinking and self-control (assessed by the Tower of London task) (F = 4.504, *p* = 0.015). Post-hoc Bonferroni t-tests revealed that this was driven by the difference in performance between ME/CFS patients and healthy controls (ME/CFS vs HC *p* = 0.012). Results for all cognitive tests are presented in Supplementary Table 2.

### Magnetic resonance spectroscopy

#### Brain MRS

PgACC/dACC/muscle data were available, respectively, for 24/23/21 ME/CFS patients, 21/17/22 long COVID patients and 24/22/23 HCs. Muscle datasets were rejected in case of two ME/CFS patients due to low data quality, and not acquired in case of one participant. In five long COVID patients dACC data were not obtained. In two of these participants pgACC data were rejected due to poor quality (first participant FWHM = 0.079, second participant SNR = 11, both > 3 SD). In case of one long COVID participant the whole dataset (pgACC, dACC and muscle data) was lost due to technical issues with data transfer, and two datasets were not analysable due to technical issues during the acquisition. Muscle datasets were rejected for two long COVID patients due to poor data quality. In two healthy controls dACC data were not obtained; muscle data from one of these participants were rejected due to low quality. All remaining spectra were of good quality and there were no between-group differences in scan quality measures FWHM and SNR (*p* > 0.05) (Table 2).

**Table 2:** Mean (SEM) absolute concentrations (mmol/kg) of brain neurochemicals corrected for cerebrospinal fluid (CSF), grey matter (GM) and white matter (WM) content in pregenual and dorsal anterior cingulate cortex (respectively, pgACC and dACC); scan quality measures (FWHM: Full Width Half Maximum, SNR: Signal To Noise Ratio); voxel content (GM: grey matter, WM: white matter, CSF: cerebrospinal fluid).

	CFS patients	Long COVID patients	Healthy controls	Univariate GLM/one-way ANOVA (F value, *p*)	Univariate GLM between two groups: ME/CFS vs HC Long COVID vs HC ME/CFS vs long COVID
pgACC					
Creatine (*n* = 24, 21, 24)	5.45 (0.18)	5.89 (0.22)	5.65 (0.19)	1.352, 0.266	N/A
					N/A
					N/A
Glutamate (*n* = 24, 21, 24)	14.66 (0.34)	14.12 (0.27)	13.78 (0.25)	2.344, 0.104	N/A
					N/A
					N/A
Glutamine (*n* = 24, 21, 24)	4.00 (0.16)	4.13 (0.19)	3.92 (0.16)	0.378, 0.687	N/A
					N/A
					N/A
Glutathione (*n* = 24, 21, 24)	1.67 (0.05)	1.42 (0.10)	1.51 (0.05)	3.373, 0.040	3.434, 0.070
					0.643, 0.427
					6.802, 0.013
Lactate (*n* = 22, 19, 22)	1.13 (0.08)	0.91 (0.06)	0.88 (0.06)	4.684, 0.013	9.586, 0.004
					0.136, 0.714
					4.031, 0.052
Total choline (*n* = 24, 21, 24)	2.16 (0.08)	2.32 (0.07)	2.16 (0.06)	1.093, 0.341	N/A
					N/A
					N/A
FWHM	0.037 (0.001)	0.039 (0.002)	0.040 (0.002)	0.533, 0.589	N/A
					N/A
					N/A
SNR	34.67 (1.06)	32.00 (1.69)	34.88 (0.99)	1.564, 0.217	N/A
					N/A
					N/A
GM (%)	64.2 (0.8)	62.0 (0.7)	63.1 (0.4)	1.997, 0.144	N/A
					N/A
					N/A
WM (%)	13.7 (0.6)	13.8 (0.6)	13.8 (0.6)	0.019, 0.981	N/A
					N/A
					N/A
CSF (%)	22.1 (0.8)	24.2 (1.0)	23.1 (0.8)	1.261, 0.290	N/A
					N/A
					N/A
dACC					
Creatine (*n* = 23, 17, 21)	5.62 (0.13)	5.99 (0.19)	5.58 (0.12)	1.490, 0.234	N/A
					N/A
					N/A
Glutamate (*n* = 23, 17, 21)	13.77 (0.22)	13.43 (0.29)	13.36 (0.18)	1.527, 0.226	N/A
					N/A
					N/A
Glutamine (*n* = 23, 17, 21)	4.03 (0.13)	3.95 (0.16)	4.12 (0.15)	0.703, 0.499	N/A
					N/A
					N/A
Glutathione (*n* = 23, 16, 21)	1.56 (0.07)	1.48 (0.03)	1.61 (0.03)	1.054. 0.355	N/A
					N/A
					N/A
Lactate (*n* = 22, 16, 17)	1.14 (0.07)	1.11 (0.08)	0.98 (0.03)	4.634, 0.014	8.618, 0.006
					2.007, 0.167
					3.089, 0.088
Total choline (*n* = 23, 17, 21)	1.99 (0.07)	1.77 (0.11)	2.20 (0.09)	8.098, 0.0008	1.625, 0.210
					17.692, 0.0002
					6.669, 0.014
FWHM	0.027 (0.001)	0.029 (0.001)	0.043 (0.015)	0.972, 0.384	N/A
					N/A
					N/A
SNR	40.91 (1.42)	38.94 (1.74)	40.90 (0.943)	0.619, 0.542	N/A
					N/A
					N/A
GM (%)	62.7 (0.6)	60.8 (0.6)	61.4 (1.0)	1.383, 0.259	N/A
					N/A
					N/A
WM (%)	20.9 (0.7)	22.4 (1.0)	19.9 (0.7)	2.200, 0.120	N/A
					N/A
					N/A
CSF (%)	16.4 (0.8)	16.8 (0.8)	18.7 (0.8)	2.377, 0.102	N/A
					N/A
					N/A

For all metabolites, there were no between-group differences in terms of CRLB (*p* > 0.05). For all metabolites, univariate general linear model (GLM) were performed adjusting for age, with two-group comparisons performed for significant results only. For other measures, one-way ANOVA was performed.

The fitting routine did not always fit all the components in all the spectra, which is reflected by the number of participants for a given metabolite in Table 2. No values were entered into the statistical analysis for these metabolites. Example spectra from the pgACC and dACC are shown in Fig. 1. There were no significant between-group differences in GM, WM and CFS content (Table 2).

##### Pregenual ACC (pgACC)

**Fig. 3:**
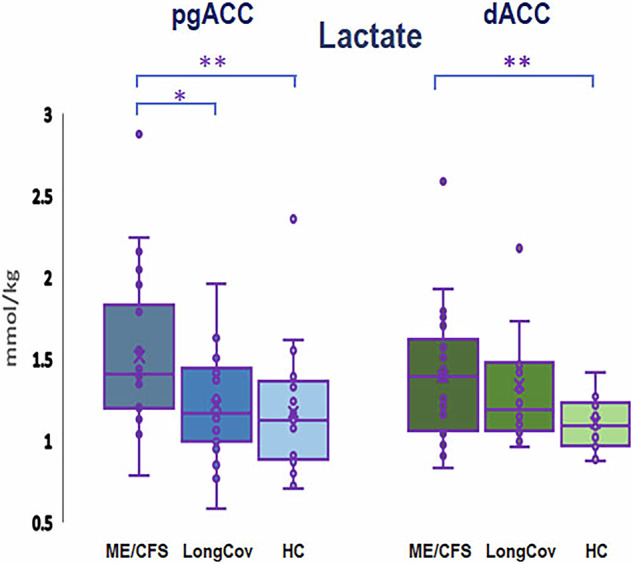
Lactate concentrations in the pregenual anterior cingulate (pgACC) and dorsal anterior cingulate (dACC) by group. In ME/CFS, lactate concentrations (in mmol/kg) were significantly higher in both pgACC (*p* = 0.004) and dACC (*p* = 0.006) as compared with healthy controls. Number of participants: pgACC, CFS - 22, long COVID - 19, HC 22; dACC, CFS - 22, long COVID - 16, HC - 17. * indicates significant results with *p* value > 0.01 and ≤ 0.05, ** indicates highly significant results with *p* velue ≥ 0.0001 and < 0.01.

The results were negative for our primary objective, i.e. replicating lower levels of creatine in ME/CFS as compared to HCs observed in our pilot 7T study. Additionally, there were no differences in creatine between long COVID patients and HCs and between the patient groups. However, univariate GLM with age as a covariate revealed other neurochemical abnormalities in the pgACC, notably in concentration of lactate (F = 4.684, *p* = 0.013) (Fig. 3). Further exploration revealed that this was driven mainly by the difference between patients with ME/CFS and HCs (F = 9.589, *p* = 0.004), with ME/CFS patients showing higher lactate levels than controls, although a trend towards significant difference between patients with ME/CFS and long COVID was also noted (F = 4.031, *p* = 0.052). Between-group differences for other neurometabolites potentially of interest in ME/CFS and long COVID, including GSH and total choline, were non-significant. MRS data and statistics are presented in Table 2.

##### Dorsal ACC (dACC)

**Fig. 4:**
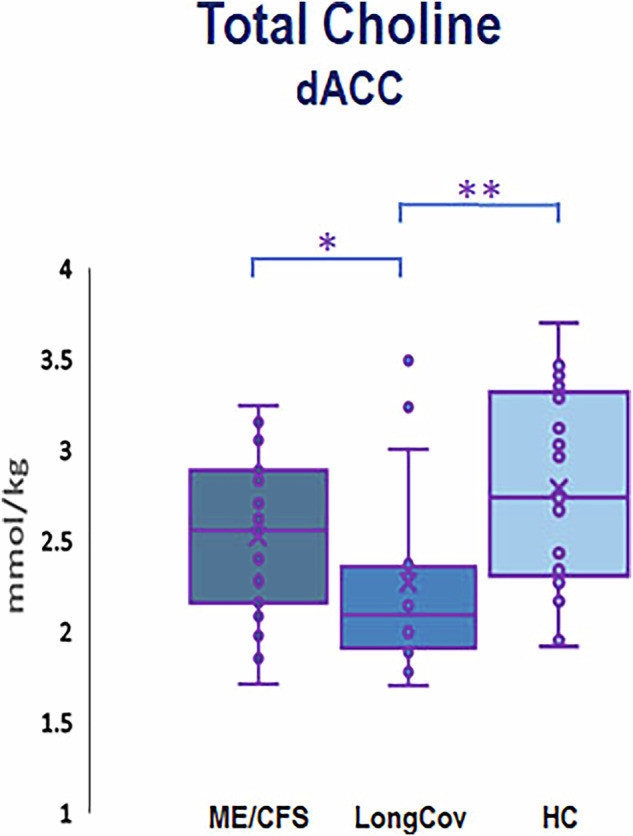
Total choline concentrations in the dorsal anterior cingulate (dACC by group). In long COVID, total choline concentrations were lower in dACC as compared with healthy controls (*p* = 0.0002). The between-group differences in pgACC were non-significant. Number of participants: CFS - 23, long COVID - 17, HC 21. * indicates significant results with *p* value 0.01 and ≤ 0.05, ** indicates highly significant results with *p* velue ≥ 0.0001 and 0.01.

In dACC, univariate GLM with age as a covariate also revealed between-group differences in the concentrations of lactate (F = 4.634, *p* = 0.014) (Fig. 3) and total choline (F = 8.098, *p* = 0.0008) (Fig. 4). In the case of lactate further analysis revealed that this was driven by the difference between patients with ME/CFS and HC (F = 8.618, *p* = 0.006), with ME/CFS patients showing higher lactate levels than controls, similar to pgACC. The difference between ME/CFS and long COVID patients was however non-significant. With total choline, the difference was driven by lower levels in long COVID compared to the HC group (F = 17.692, *p* = 0.0002); the difference between patients with ME/CFS and long COVID was also significant (F = 6.669, *p* = 0.014).

#### Muscle MRS

In the muscle, there were no statistically significant between-group differences in terms of creatine, carnitine, acetyl-carnitine, carnosine, components containing trimethylamine (TMA) group, intramyocellular lipids (IMCL), and extramyocellular lipids (EMCL). The carnosine showed a weak trend (F = 2.464, *p* = 0.094), with the highest concentration in HC (3.88 mM), intermediate in long COVID (3.03 mM) and lowest in CFS/ME (2.88 mM). MRS data and statistics are presented in Table 3.

**Table 3:** Mean (SEM) absolute concentrations (mmol/kg) of muscle biochemicals.

	CFS patients	Long COVID patients	Healthy controls	Univariate ANOVA (F value, *p*)
Carnosine (mM) (*n* = 21, 22, 21)	2.88 (0.23)	3.03 (0.39)	3.88 (0.33)	2.464, 0.094
Acetylcarnitine (mM) (*n* = 21, 22, 21)	5.28 (0.98)	6.34 (0.85)	4.00 (0.52)	2.383, 0.101
Creatine (mM) (*n* = 21, 22, 23)	31.58 (1.56)	31.89 (1.78)	27.34 (1.77)	2.189, 0.121
Trimethyl Ammonium (nM)	29.26 (6.17)	31.86 (4.55)	21.42 (2.78)	1.419, 0.250
Intra-myocellular lipids (IMCL) (% water) (*n* = 21, 22, 23)	0.5 (0.1)	1.1 (0.03)	0.9 (0.1)	1.442, 0.244
Extra-myocellular lipids (EMCL) (% water) (*n* = 21, 22, 23)	1.5 (0.2)	1.4 (0.3)	1.4 (0.2)	0.601, 0.551

For all metabolites, univariate general linear model (GLM) was performed adjusting for age, with two-group comparisons not performed as GLM results were not significant.

#### Brain metabolites and demographics/clinical scores

In ME/CFS patients, there were no correlations between fatigue scores and levels of lactate. Similarly there was no significant correlation with ratings of anxiety or depression or length of illness. Generally, there were no correlations between total choline and these measures in the Long COVID group; however, dACC total choline correlated positively with length of illness (r = 0.605, *p* = 0.01).

#### Brain metabolites and cognition

There were no significant correlations between the measures of cognition and abnormal neurometabolites in either patient group.

## Discussion

As far as we are aware, this is the first investigation using MRS at 7T to study brain neurochemistry in patients with long COVID, and the second – the first one being our pilot study [19] - in ME/CFS. Also, to the best of our knowledge, this is the first ^1^H MRS at 7T to assess muscle chemistry in either patient group. We did not confirm the findings from our pilot study of decreased creatine and GSH in pgACC in patients with ME/CFS. However, we did find in ME/CFS raised lactate concentrations in both pgACC and dACC, and in long COVID, decreased concentrations of total choline in dACC, compared to healthy controls.

The increase in lactate that we detected points towards a defect in oxidative energy metabolism, but this conclusion must be treated cautiously given the difficulty of measuring lactate in the presence of the much larger and broader macromolecule (MM) resonance at ~1.35 ppm, with the lactate signal being a small shoulder on that peak (Fig. 1). The quantification of lactate is possible because the MM components are included in the basis set and because LCModel ‘expects’ to find lactate. Despite these caveats, an increase in brain lactate in ME/CFS patients has been previously reported in 3T MRS investigations, in the ventricle CSF rather than in the brain tissue [14–17], and also in the ACC, similar to our study (the voxel in this study was placed in the area covering adjacent parts of our voxels) [13]. Taken together the findings suggest that raised brain lactate levels may be a potential biomarker of illness in some patients with ME/CFS. Further work is needed to confirm this, using a lactate editing sequence such as MEGA-sLASER.

Lactate is undoubtedly involved in energy metabolism. Initially suggested as a waste product of anaerobic glycolysis, it was later also recognized as a source of energy [12, 57, 58]. Lactate accumulation is commonly accepted as a sign of mitochondrial ill-health, indicating that mitochondria are not able to utilize either glucose or lactate as the source of energy [24]. However, the role of lactate is more complex than this [12, 58]. Lactate is formed, reductively, from pyruvate as the final reaction of glycolysis, diverting pyruvate from oxidation to acetyl-CoA which is the first step in oxidative phosphorylation. In low oxygen situations (hypoxia, ischaemia) lactate increases several-fold as its conversion from pyruvate in the lactate dehydrogenase reaction is essential to regenerate NADH needed to maintain glycolysis [59]. Thus, it is tempting to attribute an increase in lactate to an imbalance between glycolysis and mitochondrial oxidation, similar to, but less severe than hypoxia. However, as explained in detail in a recent review by Rae et al. [58] pyruvate, which is the only source of endogenous lactate in the brain, is at a crossroads of a number of metabolic pathways i.e. glycolysis, oxidative phosphorylation, anaplerosis and transamination, linking it to neurotransmitter metabolism, energy production and redox homeostasis. Changes of flux in any one of these pathways will influence pyruvate concentration and thus directly affect lactate concentration as the two are maintained close to thermodynamic equilibrium. Given this complex picture, although mitochondrial dysfunction, suggested by a number of studies [60], is a plausible reason for lactate elevations in ME/CFS, the other possibilities should not be discarded.

Increased lactate concentrations are also intriguing in the context of one of the influential hypotheses on the provenience of ME/CFS symptoms, a state of chronic, low-level neuroinflammation [61]. Neuroinflammation in ME/CFS was initially suggested by studies showing elevated levels of pro-inflammatory cytokines, and decreased anti-inflammatory cytokines, in cerebrospinal fluid from lumbar punctures in ME/CFS patients [62, 63], and further supported by a positron emission tomography (PET) study [64], which showed increased binding of a radioligand to 18 kDa translocator protein (TSPO) receptor, the expression of which increases when microglia are activated. Increased binding was present in the hippocampus, amygdala, thalamus, midbrain, pons, and cingulate cortex, although the exact area of the cingulate cortex was not defined. This is interesting in the context of our finding of increased lactate in both pgACC and dACC [9], as well as of a similar finding by Mueller at al.in their MRSI study [13]. While non-activated microglia preferentially use oxidative phosphorylation, activated microglia undergo a metabolic reprogramming, with increased glucose uptake and strengthening of anaerobic glycolysis, to increase ATP production [65]; this will lead to increased lactate concentrations. This presents an intriguing possibility that lactate could be a surrogate of inflammation, not only in ME/CFS but also other conditions. It needs to be however noted that a recent PET study [66] did not replicate Nakatomi et al. [64] findings of increased TSPO binding in ME/CFS.

We found no significant change in lactate in long COVID patients compared to healthy controls. In this they differed from ME/CFS patients. However, we did find in dACC in long COVID patients lower levels of total choline. This was unpredicted and may be a chance finding. However, in view of the recently described link between blood clots and brain fog in long COVID [67], it is of interest that choline administration has been reported to be neuroprotective in a range of animal models, and in the prevention of disseminated intravascular coagulation [68, 69]. Zhong et al. [70] reported that in post-stroke patients, the risk of cognitive impairment was inversely correlated with plasma choline levels.

The measurements in this study were taken from two voxels, positioned in two anatomically and functionally different parts of the ACC, its rostral portion, the pregenual ACC (pgACC) and in its dorsal portion, the dorsal ACC (dACC) [33]. The ACC as a whole is involved in emotional and cognitive processing and plays a crucial role in associative processing leading to the integration of information from various levels [34]. The pgACC is considered to be more ‘affective’ and involved in emotional regulation and autonomic integration, while dACC is thought to be more ‘cognitive’ and involved in conflict-monitoring, response-selection and execution [35, 36]. Relevant to this, dACC has been proposed as one of the core regions in the recently proposed ‘neurocognitive framework’, a system weighing up the costs and benefits of continued exertion in cognitive and physical tasks [71].

Difficulty thinking and mental fatigue, broadly described as ‘brain fog’ [72], is one of the most common and subjectively disturbing symptoms in both ME/CFS [73] and long COVID [74, 75]. Recent meta-analyses of neuropsychological tests showed significant declines in attention, reaction time and memory in both conditions [73, 76, 77]. Given the burden of cognitive dysfunction, a better understanding of biological underpinnings is crucial, and MRS allows a unique insight into biochemical changes linked to cognitive performance. However, our BACS test battery revealed minimal changes in cognitive performance between the patients and healthy controls. This suggest that the BACS tasks we used are not suitable for detecting cognitive dysfunction linked to complaints of brain fog in ME/CFS and long COVID.

### Muscle MRS

In the muscle, we observed a trend towards between-groups differences in carnosine, with the highest concentration in HC, intermediate in long COVID and lowest in ME/CFS. While these differences were not significant (*p* = 0.094), the trend is potentially informative of what may be happening in the muscle in fatigue related disorders. Carnosine, abundant in the skeletal muscle, has a role as a pH buffer and protects the muscle against acidosis created during anaerobic energy production, the end products of which are lactate anions and H^+^ [78]. Decreased levels of carnosine seen in our study in both ME/CFS and long COVID may contribute to patients’ experience of muscle fatigue, which has been previously associated with acidosis [79]. Other ^1^H MRS detectable muscle metabolites also did not differ significantly between the groups. Importantly, this was the case also for acetyl-carnitine, which is synthesized in the muscle from carnitine and acetyl‐CoA when mitochondrial acetyl‐CoA is abundant and exceeds its usage by the tricarboxylic acid (TCA) cycle. Acetyl-carnitine typically increases during β-oxidation, a process of energy production from fatty acids [23]. The lack of between-group differences in acetyl-carnitine in our study indicates a good usage of fatty acids as the energy source in all the groups examined. Taken together, the findings regarding carnosine and acetyl-carnitine suggest that oxidative respiration may be less efficient in both ME/CFS and long COVID but both groups of patients are able to use lipids as a source of energy. This raises an intriguing question of whether alternative sources of energy, such as ketone bodies by-passing the glycolytic pathway, might lead to increases in energy, and indeed some studies showed a reduction in fatigue in ME/CFS on a ketone diet [80]. It needs to be noted that all the measurements were done at rest. Fatigue is often more pronounced with physical activity, so there is a possibility that group differences would become evident with exercise.

When interpreting these data, it needs to be considered that biochemical abnormalities may be consequences, rather that the cause, of prolonged inactivity. In the current study there was no correlation between the abnormal neurochemical findings and the length of illness in ME/CFS. In the long COVID group there was a correlation of the length of illness with dACC and total choline (r = 0.605, *p* = 0.01). This, however, is a non-a-priori correlation, not corrected for multiple comparisons, and therefore should be viewed with caution. Useful for this discussion is a recent meta-analysis of MRS studies, which found that physical activity, rather than sedentary lifestyle, is related to higher brain lactate [81].

While our findings of neurochemical abnormalities in the pgACC and dACC, in ME/CFS and long COVID patients are intriguing, they must be viewed with caution given the limitations of the study. One of them is the small number of patients, which may lead to false positives, especially as we made no correction for multiple comparisons, given the exploratory nature of this study. The possibility that neurochemical changes in the patient group may not be specifically related to the presence of fatigue also needs to be considered. Physical health problems and diversity of medications used, including antidepressants, were difficult to control for. In particular, long COVID patients had symptoms of ill physical health as a consequence of the acute Sars-Cov-2 infection, even if not diagnosed as a separate condition (such as ongoing difficulties breathing). Medications and comorbidities are detailed in Supplementary Table 1.

Another important issue was that only people with mild to moderate symptom severity were included in the study, as participants had to be able to travel for their scan. This unfortunately means that we were unable to get insights into biological processes in patients whose symptoms were more severe, in whom the underlying pathologies might be more accentuated. Also, patients with ME/CFS commonly employ careful symptom management through planning their activities and rest. This is crucial for their well-being but might restrict what can be observed during testing, especially in cases of milder symptom severity. One potential research strategy might be accentuating the differences through functional challenges, such as exercising the muscle in the scanner (a strategy used by Finningan et al. [82] in long COVID), or measuring brain metabolites while participants perform a cognitive task or experience a physiological stimulus such as pain or photostimulation [83].

Our results in ME/CFS differed from the results of our pilot study. In particular, we were unable to show a decrease in creatine, despite using the same design and MRS methodology, including the use of the same scanner and MRS sequences. Analysis method however differed (QUST in jMRUI vs LCModel), which could contribute to observed differences. Difficulties in replicating findings is unfortunately the case of many MRS studies, in both ME/CFS and other conditions. One potential explanation is group heterogeneity, in this case an inclusion of participants with different biological backgrounds despite similar clinical presentations, which might lead to a decrease in power to detect changes, especially if these are subtle.

Heterogeneity is indeed an important issue to consider in designing future studies. Detailed phenotyping of patients, which includes not only questionnaires but also other biological measures, is necessary to better understand the links between the biology of ME/CFS and the clinical presentation. A number of phenotypic elements should be carefully assessed, including neurological and cognitive symptoms (such as impairment of concentration and short-term memory, perceptual and sensory disturbances, sensory and emotional overload phenomena), autonomic manifestations (such as orthostatic intolerance, palpitations, gastro-intestinal symptoms), neuroendocrine manifestations (such as loss of thermostatic stability, intolerances of extremes of heat and cold, worsening symptoms with stress), and immune manifestations (such as tender lymph nodes, recurrent sore throat or flu-like symptoms, general malaise (flu-like feelings of being ill and feverish). This complex assessment could help to cluster patients into subgroups that can be better explained by specific biological dysfunctions.

Possible heterogeneity may also mean that one single biological measure may not be feasible as a marker for ME/CFS. Relevant to this study is the fact that increased lactate has been found in other neurodegenerative conditions, such as multiple sclerosis. It is likely that a brain biomarker will need to be combined with additional measures, such as blood-based lactate or inflammatory measures, to be more specific to ME/CFS or its subtypes. The above-mentioned approach of functional MRS may also be helpful as it may reflect better the immediacy of fatigue and PEM, characteristic for ME/CFS. This will need future research.

An observation of potential importance for future research and treatment development is that the ME/CFS and long COVID groups tested in this study differed in terms of their brain neurochemistry measured by MRS. It is therefore possible that the underlying neurobiological mechanisms, while leading to similar clinical presentation of fatigue and brain fog, may differ between these groups. While this needs to be verified with future research, an important implication is that patients with ME/CFS and those with fatigue in the course of long COVID should not be studied as a single group, at least until the mechanisms are better understood.

## Supplementary information

Supplementary material

## Data Availability

The datasets generated and/or analysed during the current study are available from the corresponding author on reasonable request.
