# Supplementary material for: Brain and muscle chemistry in myalgic encephalitis/chronic fatigue syndrome (ME/CFS) and long COVID: a 7T magnetic resonance spectroscopy study
Source: Mol Psychiatry. 2025 Jul 12;30(11):5215–26. doi: 10.1038/s41380-025-03108-8 (PMC12532701; doi:10.1038/s41380-025-03108-8)
Supplement: Supplementary file 1 — Supplementary material [file 41380_2025_3108_MOESM1_ESM.docx]

Supplementary Table 1. The list of prescription medications used by individual participants and comorbidities.

|  | **Medications/Supplements** | **Medical conditions** |
| --- | --- | --- |
|  |  |  |
| **ME/CFS patients** |  |  |
| 1 | - | - |
| 2 | Levothyroxine | Hashimoto’s disease (T3 and T4 within normal range on treatment) |
|  | - |  |
|  | - | - |
| 3 | Nortyptyline 70mg (against pain), vit D, omeprazole 20mg | - |
| 4 | Omeprazole 20mg, sertraline 50mg, amitriptyline 20mg (against pain), vit D, vit B12, Mg | Fibromyalgia |
| 5 | - | - |
| 6 | - | - |
| 7 | Candesartan, vit D | Hypertension |
| 8 | - | Cow’s milk allergy |
| 9 | Levothyroxine, vitamin D, HRT | Hashimoto’s disease (T3 and T4 within normal range on treatment) |
| 10 | Supplements | - |
| 11 | - | - |
| 12 | - | - |
| 13 | Loratidine, chlorphenamine, salbutamol (all PRN, not taken at the time of testing) | Hayfever |
| 14 | Sertraline 150mg, salbutamol PRN | Generalized anxiety disorder, mild asthma |
| 15 | Fluoxetine 20mg, omeprazole, fexofenadine, combined contraceptive pill, vit D, vit B comb, vit C | Ehler-Danlos syndrome, stomach discomfort, fibromyalgia |
| 16 | Omeprazole, norethindrone, tramadol PRN, contraceptive coil Mirena (evonorgestrel-releasing intrauterine system) | Endometriosis |
| 17 | - | - |
| 18 | Duloxetine 60mg, melatonin 2mg, Mg, Ca, vit D3, multivitamine | Hayfever |
| 19 | HRT, contraceptive coil Mirena (evonorgestrel-releasing intrauterine system) | - |
| 20 | Fexofenadine, famotidine, ibuprofen, paracetamol (all PRN) | - |
| 21 | Beclometasone | Hayfever, mild asthma |
| 22 | Melatonin 1mg | - |
| 23 | - | - |
| 24 | Pregabalin 150mg, duloxetine 60mg, lansoprazole, HRT | Fibromyalgia |
| **Long COVID patients** |  |  |
| 1 | Citalopram 20mg | MDD, controlled with citalopram |
| 2 | - | - |
| 3 | Amlodipine, sertraline | Hypertension, osteopenia, depression and anxiety |
| 4 | - | - |
| 5 | AirFlusal (25 micrograms salmeterol and 125 micrograms fluticasone) inhaler, amitriptyline (for pain and sleep), lansoprazole, vit D | Post-COVID asthma |
| 6 | Zinc, niacin, vit C, vit D, vit E, Mg, Omega 3, folate, black seed oil, collagen, CBD oil, olive leaf extract | - |
| 7 | Ibuprofen, Ca, Mg, vit D, vit C, vit B, zinc |  |
| 8 | Fluoxetine 20mg, ivabradine 10mg | Asthma, anxiety |
| 9 | AirFlusal (25 micrograms salmeterol and 125 micrograms fluticasone) inhaler; pregabalin |  |
| 10 | Sumatriptan PRN, multivitamins |  |
| 11 | - |  |
| 12 | Ivabradine; loratidine; avamys nasal spray fFluticasone furoate) | Hayfever, seasonal rhinitis |
| 13 | - | - |
| 14 | - | - |
|  | - | - |
| 15 | Citalopram, omeprazole | Hayfever |
| 16 | - | - |
| 17 | Amitriptyline 30mg for sleep | - |
| 18 | - | - |
| 20 | Montelukast, fostair 200, amamys nasal spray, cetirizine, vit D, forceval, ferrograd | Asthma, hayfever, hereditary fructose intolerance |
| 21 | Vit B6, B12, Fe, folic acid | - |
| 22 | Loratidine, Mg, multivitamine, probiotics | - |
| 23 | Fostair 200, salbutamol PRN | Asthma |
| 24 | Rotigotine, melatonine, antihistamines, HRT | Restless legs syndrome |
| 25 | - | - |
| **Healthy Controls** |  |  |
| 1 | Nexplanone (contraceptive etonogestrel implant) 68 mg | - |
| 2 | HRT | - |
| 3 | Salbutamol PRN | Mild asthma |
| 4 | Candesartan | Hypertension |
| 5-8 | Combined contraceptive pill | - |
| 9-11 | Contraceptive coil Mirena (evonorgestrel-releasing intrauterine system) | - |
| 12-24 | - | - |

Supplementary Table 2. Cognitive tasks (Brief Assessment of Cognition in Schizophrenia (BACS) scores. Values represent numbers or mean (SEM). Independent samples t-tests were performed only for significant one-way ANOVA.

|  | ME/CFS patients (24) | Long COVID patients  (25) | Healthy controls (24) | Statistics: one-way ANOVA (F, p) | Independent samples t-tests  ME/CFS vs HC  Long COVID vs HC  ME/CFS vs long COVID |
| --- | --- | --- | --- | --- | --- |
|  |  |  |  |  |  |
| Composite z-score | 0.33 (0.22) | 0.51 (0.20) | -0.005 (0.16) | 1.904, 0.158 | N/A |
|  |  |  |  |  | N/A |
|  |  |  |  |  | N/A |
| Verbal memory (word list learning) | -0.41 (0.22) | -0.18 (0.19) | -0.36 (0.23) | 0.338, 0.714 | N/A |
|  |  |  |  |  | N/A |
|  |  |  |  |  | N/A |
| Working memory (digital sequencing) | -0.24 (0.21) | 0.20 (0.21) | -0.07 (0.22) | 1.072, 0.349 | N/A |
|  |  |  |  |  | N/A |
|  |  |  |  |  | N/A |
| Verbal fluency (oral word association) | 0.60 (0.29) | 1.05 (0.32) | 0.34 (0.22) | 1.673, 0.196 | N/A |
|  |  |  |  |  | N/A |
|  |  |  |  |  | N/A |
| Attention and speed of information processing (symbol coding) | 0.18 (0.23) | -0.10 (0.17) | -0.14 (0.16) | 0.775, 0.465 | N/A |
|  |  |  |  |  | N/A |
|  |  |  |  |  | N/A |
| Executive function (Tower of London) | 1.04 (0.29) | 0.60 (0.12) | 0.27 (0.12) | 4.504, 0.015 | 2.629, 0.012 |
|  |  |  |  |  | 1.957, 0.057 |
|  |  |  |  |  | 1.568, 0.125 |
| Motor function (token motor task) | 114.1 (7.4) | 101.4 (6.5) | 101.2 (6.0) | 1.126, 0.331 | N/A |
|  |  |  |  |  | N/A |
|  |  |  |  |  | N/A |
|  |  |  |  |  | N/A |
|  |  |  |  |  | N/A |
